# Ancillary ICU Care in Patients with Acute Brain Insults

**DOI:** 10.5005/jp-journals-10071-23193

**Published:** 2019-06

**Authors:** Kapil Dev Chhabra, Mandeep Singh

**Affiliations:** 1,2 Department of Critical Care Medicine, Maharaja Agrasen Hospital, New Delhi, India

## Abstract

**How to cite this article:** Chhabra KD, Singh M. Ancillary ICU Care in Patients with Acute Brain Insults. Indian J Crit Care Med 2019;23(Suppl 2):S147–S150.

Acute brain injury (ABI) is a common entity to deal with in neurocritical care ICU's. They result either from direct brain injury due to various disease processes namely acute ischemic strokes (AIS), intracerebral Hemorrhages (ICH), traumatic brain injury (TBI), anoxic encephalopathy and CNS infections. The continuum of direct brain injuries are indirect or secondary brain injuries which are preventable and treatable. The mechanisms behind such injuries are brain edema, ischemic cascade/cellular injury, cerebral perfusion/autoregulation abnormalities, and cerebrospinal fluid/intracranial pressure derangement. But as these critically ill patients require long ICU stay, they are receptive to various medical complications. These clinical entities affect the outcome significantly. These complications are easily preventable and treatable with appropriate interventions. These medical complications can be classified as infective and non-infective complications.

## INFECTIVE COMPLICATIONS

Infective complications are common in these patients as they require central venous catheters, arterial cannulation, urinary catheters, intraventricular catheters so on and so forth, for monitoring and therapeutic interventions. These devices are susceptible to various infectious complications. Dettenkofer et al found that, 113 such infections occurred in 545 neurosurgical patients.^1^ In a study by O’Shea et al, 21 out of 73 patients developed 22 infections after a mean ICU stay of 5 days. Out of 22, 9 were respiratory infections, 7 urinary tract infections, 4 central nervous system infections, and wound and skin infections (one each). The responsible organisms included *Pseudomonas* (7/21), *Acinetobacter* (3/21), *E. coli* (2/21), *Enterobacter* (2/21), and *Klebsiella* (2/21), and one each of *Staphylococcus aureus*, methicillin resistant *Staphylococcus aureus*, coagulase negative *Staphylococcus*, group D *Streptococcus* and bacteroides.^2^ These infections are easily preventable with implementation of appropriate infection control practices. Hand hygiene is the most important and integral part of all infection control practices. Moreover these infections are difficult to treat as isolates are often multidrug resistant being ICU-acquired.

Intracranial pressure monitoring is an important intervention in neurology and neurosurgical ICUs. One of the methods is to place catheter in ventricles i.e. external ventricular drain (EVD). It carries an infection rate to the range of 4.1–10%. Khan et al in a retrospective study found intraparenchymal devices carry a lower risk of infection (0.6%), than percutaneous ventriculostomies (4.4%).^3^ High level of suspicion in patients with new onset fever, leucocytosis, any new organ dysfunction with such devices in situ leads to early diagnosis. After diagnosis, catheter removal or change, along with cerebrospinal fluid culture is extremely important in the management. The administration of appropriate antibiotics for optimal duration is standard of care.

The other nosocomial infections such as urinary tract infection (UTI), ventilator associated pneumonia (VAP) in patients who received invasive mechanical ventilation and blood stream infections (BSI) are also common in these patients.

## NON INFECTIVE COMPLICATIONS

### Electrolyte Disturbances

The most common electrolyte abnormality is hyponatremia in neurocritical care ICU. The most common etiologies responsible for hyponatremia are syndrome of inappropriate antidiuretic hormone secretion (SIADH) and cerebral salt wasting syndrome (CSWS). Differentiating between these two entities are important as management of these two entities is entirely different. The occurrence of SIADH is common with various diseases such as malignancies including brain tumors and lung cancer (non-small cell lung carcinoma), ICH, TBI. Use of several drugs (such as chlorpropamide, carbamazepine, cyclophosphamide, and vincristine) can lead to SIADH. Its diagnosis can be considered in patients with hyponatremia (serum sodium levels <135 mEq/dL) with hypotonic serum osmolality (<295 m Osm/kg), urinary sodium >20 mEq/dL and urine osmolality more than serum osmolality. The patient fluid status in SIADH is euvolemia. Its management in symptomatic patient is hypertonic saline (3% NaCl) with fluid restriction. In asymptomatic patients, only fluid restriction is appropriate. The cerebral salt wasting syndrome (CSWS) also occurs with a huge list of diseases namely TBI, SAH, and intracranial infections like tubercular meningitis.^4^ Its diagnosis requires hyponatremia in the background of low serum osmolality, hypovolemia and urine spot sodium >20 mEq/dL. Its management in symptomatic patients requires 3% NaCl with fluid therapy.

Hypernatremia is a next common electrolyte abnormality in this subset of patients. Its association is common with TBI. There are variations among different studies regarding threshold value to define hypernatremia but in a study by Aiyagari et al, serum sodium value 150 mEq/L was taken as upper limit of normal and value above it was considered as hypernatremia.^5^ The mortality increases with increasing intensity of hypernatremia, but only severe hypernatremia (serum sodium >160 mEq/L) was independently associated with increased mortality. The causes of hypernatremia are multifactorial. Hypernatremia may occur either from sodium gain or free water loss. Many a times it may be induced by the clinician as ICP lowering measure. So to determine the underlying cause is extremely important. Diabetes insipidus (DI) is a common cause for hypernatremia and as well as a risk factor for mortality in patients with TBI.

### Hypokalemia

Gariballa et al. in their study, found that hypokalemia was associated with increased risk of death, which was independent of age, stroke severity, hypertension or smoking history.^6^ In neurocritical care ICU patients with polyuria secondary to diabetes insipidus and patients on diuretics have the tendency to develop symptomatic hypokalemia. The treatment of hypokalemia is same as in other patients. In patients with electrocardiographic changes patient should be treated with potassium chloride infusion (KCL) with regular monitoring of serum potassium to achieve target levels of 4 mEq/L. The other electrolytes need monitoring are calcium, magnesium and phosphates. The hypomagnesemia is particularly important in case of refractory hypokalemia.

### Glycemic Management

Hyperglycemia is a common abnormality that significantly affects the outcome in acute brain injury patients. The mechanism underlying the harmful effect of hyperglycemia is multifactorial: free radical formation, triggering of necrotic and apoptotic pathway of cell death etc. Hypoglycemia is equally hazardous as glucose is energy source to neuronal cells. There is a U-shaped relationship between blood glucose and neurological outcome. Thus both hyper and hypoglycemia are bad for brain. Kramer et al, in a metanalysis of 16 RCTs (1248 neurocritical care patients) found that with intensive insulin therapy targeting glucose levels 70–140 mg /dL; there was no difference in mortality when compared with conventional group targeting blood glucose levels of 144–300 mg/dL. The tight glycemic control was associated with more episodes of hypoglycemia and statistically non-significant higher mortality.^7,8^ The less aggressive blood glucose control is advantageous in comparison to very tight blood glucose control.

### Nutrition Support

Nutrition support is an essential part of care in patients in the neuro-critical care unit. Nutritional requirement in this subset of patients is determined by several factors such as baseline nutritional status, severity of illness, temporal progression of disease, mechanical ventilation, sedatives/paralyzing agents, neurosurgical procedure etc.^9^ The ICP (intracranial pressure) lowering measures significantly decrease nutritional requirement, on the other hand patients with TBI and post neurosurgical patients are hypercatabolic hence their nutritional requirements are high. The course of disease can be classified into three phases acute, chronic and recovery, but these phases considerably overlap, and nutritional requirement also varies.^10^ A personalized approach to individual patient regarding nutritional dosage and timing is extremely important. Nutritional risk stratification can be done by numerous tools such as weight/body mass index, subjective global assessment, nutric (nutritional risk in critically ill) score, NRS (nutrition risk score) 2002, predictive equations like Harris-benedict and Penn state equation, and indirect calorimetry. Indirect calorimetry is the gold standard.

The components of nutrition include carbohydrates, fats and proteins. The calories come from carbohydrates and fats. The calorie requirement can be calculated as 25–35 kcal/kg/day. The protein requirement ranges from 1.2 to 2 g/kg actual body weight/ day. In obese patients, protein 2 to 2.5 g/kg of ideal body weight/ day should be provided.

There are two routes enteral and parenteral route to provide nutritional support. The enteral route is more physiological and helps to maintain the gastrointestinal tract mucosal integrity which eventually protect the patient from translocation of gut bacteria. Both ASPEN (American society of parenteral nutrition and enteral nutrition) and ESPEN (European society of parenteral and enteral nutrition) recommend enteral route as a preferred route to provide nutritional support and to start feeding within 24–48 hours of ICU admission.^11,12^ In patients with high risk of aspiration, naso-jejunal route can be utilized for enteral feeding. Enteral route is associated with reduced infectious complications as compared to parenteral route.

If enteral route cannot be used to provide nutrition, it is recommended to use parenteral route with little differences in two guidelines. ASPEN guidelines recommend parenteral nutrition to be start after 7 days of ICU stay if enteral route cannot be utilized. In comparison ESPEN guidelines recommend to start parenteral nutrition within 3–7 days duration if enteral nutrition is contraindicated.^11,12^

In a study by Arabi et al, comparing underfeeding (40–60% of daily calories requirement) and standard feeding (70–100% of daily calories requirement) with normal protein requirement, there was a a non-significant trend toward reduced mortality in underfeeding group with no difference in 90 days mortality, hospital mortality, or ICU acquired infections.^13^

### Venous Thromboembolism Prevention

The patients in neuro critical care ICU are vulnerable to venous thrombo embolism (VTE) because of prolonged ICU-LOS, immobilization, scepticism regarding pharmacological anticoagulation in hemorrhagic strokes and traumatic brain injury, spinal cord injury (SCI) etc.^14^ The pathophysiological mechanism of VTE encompasses abnormality in Virchow triad i.e. endothelial injury, venous stasis and hypercoagulability. VTE encompass both deep venous thrombosis (DVT) and pulmonary embolism (PE). Misra et al, found a lower rate of DVT in neurosurgical patients who were given DVT prophylaxis with 5000 IU of heparin twice daily as compared to intermittent compression devices (ICD).^15^ Prevention is the mainstay of management. Pharmacological prophylaxis is the most preferred way of prevention.^16^ In traumatic brain injury (TBI), patients without DVT prophylaxis, the incidence of DVT is upto 54% and with sequential compression devices alone, it is 25%.^17,18^ VTE risk escalates with the severity of TBI. Low molecular weight heparin (LMWH) or low dose unfractionated heparin (UFH) in combination with mechanical prophylaxis should be considered. Low molecular weight heparins (enoxaparin) are preferred pharmacological agents for DVT prophylaxis in spinal cord injury (SCI). In acute spinal cord injury (SCI) Slavik et al, showed Dalteparin 5000 units subcutaneous route once daily was non-inferior to enoxaparin 30 mg twice daily.^19^ Maxwell in a study revealed no benefit of prophylactic IVC filter in patients with spinal cord injury.^20^

### Tracheostomy Care

Tracheostomy is a common procedure in neurological critically ill patients, as often they have issues with poor neurological status, inability in clearing secretions, neuromuscular weakness and thus requirement of prolonged invasive mechanical ventilatory support. So, tracheostomy is an important procedure in this subset of patients. The timing of tracheostomy is still a matter of debate. Early tracheostomy facilitates weaning, oral care, early mobilization and possibly reduces incidence of VAP and mortality. ^21^ But in a landmark trial by Young, no mortality benefit was seen with early tracheostomy.^22^ In our practice we prefer early tracheostomy where we are anticipating prolonged mechanical ventilation. There are two types of tracheostomy procedures common in critical care - surgical tracheostomy and percutaneous dilatational tracheostomy. The percutaneous tracheostomy (PCT) can be done easily at bedside, is more cost effective, requires small horizontal incision and uses seldinger technique under bronchoscopy guidance.^23^ The post procedure care includes stoma care with regular inspection for any infection and dressing change, proper suctioning with aseptic precautions. The long-term complications include tracheal or subglottic stenosis, tracheo-esophageal fistula and tracheomalacia.

### Bedsore Prevention

Bed sores (or decubitus ulcers) is one of the most devastating complications in critically ill patients and reflects poor quality of care. The bedsore development depends on four pathophysiological mechanisms namely pressure, shearing force, friction and moisture. It occurs predominantly on bony prominences as they are sites for increased local pressure which hampers the tissue perfusion. It starts with small lesion and progresses in hours to days through four stages characterized by worsening necrosis, infections and loss of tissue. The patients in neurocritical care specifically are more vulnerable to develop this complication because of long ICU stay, prolonged immobilization, and poor nutritional status. Fife et al, in 186 neurocritical care patients found that 23 of 186 patients developed at least one pressure ulcer after ICU stay of mean 6.4 days. Braden score of < 16 along with low body mass index (BMI) are the important risk factors for pressure sore occurrence.^24^ Minjuan et al found that a mean Braden score of 11.2 was associated with 31.4% incidence of bed sores.^25^ The bed sores add significantly to the morbidity, duration of ICU-LOS and increased cost of care.

In the management of bed sore, the famous Erasmus quote “prevention is better than cure” is the best treatment because once bedsore develops, it progresses very quickly through four stages unless appropriate steps are taken in a timely manner. Treatment should target first in eliminating the local pressure by routine scheduled change in position through appropriate ICU protocols, debridement of necrotic tissue, optimal nutrition, treating other factors which hinder the wound healing like infections such as underlying osteomyelitis.

## CONCLUSION

ICU care for patients with acute brain insult is different from other critically ill patients due to several reasons. Infection control practices including hand hygiene and care for indwelling catheters and ICP monitoring catheters with both insertion and maintenance bundles of care are extremely important to prevent infections. Early identification along with removal or change of device and appropriate antimicrobial for adequate duration is required. Electrolyte abnormalities are common in this subset of patients especially hyponatremia and hypernatremia. Identifying the etiology early is essential to correct the abnormalities. Early differentiation between SIADH and CSW syndrome is important as their management is different. Other electrolytes should be measured and corrected accordingly. Glucose management is important as both hyper and hypoglycemia have detrimental effects on brain tissue. Less aggressive approach is recommended to target blood glucose between 140 mg/dL and 180 mg/dL. Nutritional support should be considered early in the course of disease. Enteral route is the preferred route and personalized dose of feed should be consider as per nutritional assessment of individual patient. Venous thromboembolism is a common occurrence, proper risk assessment and initiation of DVT prophylaxis (using a combination of mechanical devices and pharmacological agents depending on the risk of bleeding). LMWH is the preferred agent. Early tracheostomy facilitates weaning while allowing clearance of pulmonary secretions. Bed sore significantly add to the morbidity and mortality to these subsets of patients. Prevention is the most important management of bed sore.
